# ASIC3: A Lactic Acid Sensor for Cardiac Pain

**DOI:** 10.1100/tsw.2001.254

**Published:** 2001-10-05

**Authors:** D.C. Immke, E.W. McCleskey

**Affiliations:** Vollum Institute, Oregon Health & Science University, Portland, OR 97201-3098, USA

**Keywords:** acid-sensing ion channels, angina, cardiac pain, ischemic pain, vasoocclusive pain, lactic acidosis

## Abstract

Angina, the prototypic vasoocclusive pain, is a radiating chest pain that occurs when heart muscle gets insufficient blood because of coronary artery disease. Other examples of vasoocclusive pain include the acute pain of heart attack and the intermittent pains that accompany sickle cell anemia and peripheral artery disease. All these conditions cause ischemia – insufficient oxygen delivery for local metabolic demand — and this releases lactic acid as cells switch to anaerobic metabolism. Recent discoveries demonstrate that sensory neurons innervating the heart are richly endowed with an ion channel that is opened by, and perfectly tuned for, the lactic acid released by muscle ischemia[1,2].

Several chemicals contribute to chest pain[3]. Bradykinin and adenosine have received the most experimental attention, whereas lactic acid received relatively little until recently. The reason is that the acidosis that accompanies a heart attack seems so trivial. At rest, blood pH is about 7.4; upon occlusion of a coronary artery, it quickly drops to 7.0, but even after much time, extracellular pH never drops below about 6.7[4]. Neutral pH doesn't seem like much of a stimulus, particularly since it can be reached during systemic acidosis, or even by breathing carbon dioxide, neither of which causes chest pain. Pan, Longhurst, and colleagues confronted this paradox with experiments in whole animals in which they record from cardiac sensory axons while occluding coronary arteries and measuring pH on the cardiac surface[5]. The sensory axons start firing action potentials in time with the drop in pH, and most importantly, the firing frequency is diminished by over 50% if a high concentration of pH buffer bathes the external cardiac surface. Nevertheless, if the animal breathes carbon dioxide until pH drops to a similar level as that reached by vascular occlusion, no increase in firing frequency occurs. Pan et al. suggest that the relevant difference between hypercapnia (the carbon dioxide) and ischemia may be the form of the acid: lactic acid in ischemia vs. carbonic acid in hypercapnia. In support of this, they show that sensory axons fire when lactic acid is applied to the heart until pH drops to 7.0, but not when other forms of acid generate the same pH change.

We now have demonstrated a molecular lactic acid sensor on sensory neurons that innervate the heart[2]. Termed ASIC3 — acid sensing ion channel #3 — it is one of five ASICs known in rats. Ion channels, the molecules that underlie bioelectricity, form transmembrane pores of atomic dimensions that selectively allow particular ions to flow, thereby generating electric current. In the case of ASICs, the pore selects $Na^+$ and it opens when the extracellular pH drops, thereby diminishing the cell's membrane potential and triggering action potentials when the media becomes acidic. ASICs were discovered by Krishtal and coworkers in the early 1980s using electrical recordings on sensory neurons[6]. In the mid 1990s, Lazdunski and coworkers demonstrated through expression of cloned channels that ASICs are a branch of a larger family of small, $Na^+$-selective ion channels[7]. In rats, ASIC3 is found only in sensory neurons[8].

Each neuron in a sensory ganglion mediates a particular sensation from a particular spot in the body. Seeking to ask whether neurons that mediate ischemic pain differ from those that mediate other sensations, we fluorescently labeled neurons that innervate the heart so they could be distinguished from others. The heart is unusual in that pain is the only conscious sensation that arises from it, and the pain can be caused only by ischemia; thus, its sensory neurons are a nearly pure population specialized for sensing ischemic pain. The labeled cardiac sensory neurons have a unique phenotype: they all have grossly high levels of $Na^+$-selective, acid-evoked current[9]. To find which, if any, of the five cloned ASICs mediated the current, we expressed each of the clones in COS7 cells and compared their functional properties to the native currents. Only ASIC3 matches the native channel in all respects[1]. Sensitivity to pH may be the most critical property that is unique to ASIC3. It is half-maximally activated at pH 6.7 and gives substantial currents at pH 7.0. Its activation curve is remarkably steep: with a Hill coefficient of 4, it is essentially 4 times more sensitive than a pH electrode in that range of pH that occurs during coronary artery occlusion. This makes ASIC3 the most sensitive biological detector of extracellular pH. Nevertheless, it becomes even more sensitive in the presence of lactate. Current evoked by pH 7.0 is increased 60% in the presence of 15 m*M* lactate[2], a concentration that occurs in muscle during intense exercise. Lactate acts by subtly decreasing extracellular divalent ions, and this shifts the activation curve, allowing the channel to open at slightly higher pH.

In summary, ASIC3 expresses at extraordinarily high levels on sensory neurons that innervate the heart, and its sensitivity to lactate and small changes in pH perfectly tune it to respond to the lactic acidosis that occurs when a coronary artery is occluded. Thus, it would seem that a selective pharmacological inhibitor of ASIC3 should suppress cardiac pain and perhaps other kinds of vasoocclusive pain. But do we want to block cardiac pain? We never want to conceal cardiac pain so well that patients are unaware of heart attack and fail to seek treatment. But this may be a false concern. Like all pain, cardiac pain has multiple signaling paths, making it unlikely that blockade of any single molecule could conceal a massive stimulus such as a heart attack. A drug that only mildly suppresses heart attack pain might significantly relieve chronic angina. Moreover, cardiac pain needs to be relieved because it is, in itself, dangerous. It triggers a powerful sympathetic reflex that releases adrenaline onto the heart, increasing cardiac pace and force at a time when the heart is already getting insufficient oxygen. The excess adrenaline triggers cardiac arrhythmias, and this, rather than ischemic damage itself, is the usual cause of heart attack death[10]. Increased sympathetic activity also accompanies angina, causing excess cardiac metabolic activity and promoting hypertrophy and heart failure[11]. Inhibition of the sensory arm of this counterproductive reflex seems certain to be valuable, and ASIC3 appears to be a major sensory molecule for it.
